# Generation and analysis of novel *Reln‐*deleted mouse model corresponding to exonic *Reln* deletion in schizophrenia

**DOI:** 10.1111/pcn.12993

**Published:** 2020-03-05

**Authors:** Masahito Sawahata, Daisuke Mori, Yuko Arioka, Hisako Kubo, Itaru Kushima, Kanako Kitagawa, Akira Sobue, Emiko Shishido, Mariko Sekiguchi, Akiko Kodama, Ryosuke Ikeda, Branko Aleksic, Hiroki Kimura, Kanako Ishizuka, Taku Nagai, Kozo Kaibuchi, Toshitaka Nabeshima, Kiyofumi Yamada, Norio Ozaki

**Affiliations:** ^1^ Department of Neuropsychopharmacology and Hospital Pharmacy Nagoya University, Graduate School of Medicine Nagoya Japan; ^2^ Brain and Mind Research Center Nagoya University Nagoya Japan; ^3^ Department of Psychiatry Nagoya University Graduate School of Medicine Nagoya Japan; ^4^ Department of Pharmacology Nagoya University Graduate School of Medicine Nagoya Japan; ^5^ Center for Advanced Medicine and Clinical Research Nagoya University Hospital Nagoya Japan; ^6^ Institute for Advanced Research Nagoya University Nagoya Japan; ^7^ Advanced Diagnostic System Research Laboratory, Graduate School of Health Sciences Fujita Health University Toyoake Japan

## Abstract

**Aim:**

A Japanese individual with schizophrenia harboring a novel exonic deletion in *RELN* was recently identified by genome‐wide copy‐number variation analysis. Thus, the present study aimed to generate and analyze a model mouse to clarify whether *Reln* deficiency is associated with the pathogenesis of schizophrenia.

**Methods:**

A mouse line with a novel *RELN* exonic deletion (*Reln*‐del) was established using the CRISPR/Cas9 method to elucidate the underlying molecular mechanism. Subsequently, general behavioral tests and histopathological examinations of the model mice were conducted and phenotypic analysis of the cerebellar granule cell migration was performed.

**Results:**

The phenotype of homozygous *Reln*‐del mice was similar to that of *reeler* mice with cerebellar atrophy, dysplasia of the cerebral layers, and abrogated protein levels of cerebral reelin. The expression of reelin in heterozygous *Reln*‐del mice was approximately half of that in wild‐type mice. Conversely, behavioral analyses in heterozygous *Reln*‐del mice without cerebellar atrophy or dysplasia showed abnormal social novelty in the three‐chamber social interaction test. *In vitro* reaggregation formation and neuronal migration were severely altered in the cerebellar cultures of homozygous *Reln*‐del mice.

**Conclusion:**

The present results in novel *Reln*‐del mice modeled after our patient with a novel exonic deletion in *RELN* are expected to contribute to the development of reelin‐based therapies for schizophrenia.

Reelin is a large extracellular matrix protein expressed in many brain regions.^1^ During developmental stages, reelin is secreted primarily by Cajal–Retzius cells and plays a crucial role in neuronal migration and layer formation in the cerebral cortex^2^, ^3^; it is also required in cerebellar development.^4^ In the adult brain, reelin is produced by GABAergic interneurons and contributes to synaptic plasticity, dendritic morphology, and cognitive function.^5^, ^6^, ^7^ Previous studies reported lissencephaly and cerebellar hypoplasia in patients carrying *RELN* mutations; this phenotype is similar to that observed in *reeler* mice.^8^, ^9^ The full‐length reelin protein is approximately 400 kDa in size, with eight repeated domains known as reelin repeats and is cleaved by proteases, such as disintegrin and metalloproteinase, with thrombospondin motifs 3 at two specific sites^10^: one site between reelin repeats 2 and 3 (N‐t site) and another site between reelin repeats 6 and 7 (C‐t site). In previous studies, two *Reln*‐mutant mice were used to evaluate reelin function. Jackson *Reln* homozygous mice (Relnrl‐J) have a 150‐kB genomic deletion, whereas Orleans *Reln* homozygous mice (Relnrl‐Orl) produce a transcript with a 220‐bp deletion.^11^, ^12^ Reelin protein is not produced in Relnrl‐J mice,^5^ whereas Relnrl‐Orl mice produce a non‐secreting protein partially cleaved at the C‐terminal.^13^, ^14^, ^15^ Little reelin signaling induced reversed cortical layering and cerebellar atrophy in both Relnrl‐J and Relnrl‐Orl mice. Furthermore, heterozygous Relnrl‐J (Relnrl‐J/+) mice showed memory dysfunction,^5^, ^16^ anxiety, and pre‐pulse inhibition (PPI). Additionally, we recently reported that heterozygous Relnrl‐Orl (Relnrl‐Orl/+) mice showed dysfunction in sociability, locomotor coordination, and anxiety.^17^ Reelin plays an important role in the cortex and hippocampus, which contribute significantly to memory formation and retention.^7^, ^16^, ^18^

Genetic studies suggest that *RELN* is associated with psychiatric disorders, including schizophrenia and autism spectrum disorder.^19^, ^20^ Specifically, rare variants of *RELN*, including *de novo* or rare missense variants and an exonic deletion of *RELN*, were identified as risk factors for schizophrenia.^21^, ^22^, ^23^ Our recent genome‐wide copy‐number variation analysis using a high‐resolution array in a cohort comprising mainly Japanese subjects identified a schizophrenic patient with a novel exonic deletion in *RELN*.^24^ We further confirmed that the relative amount of reelin in serum was lower in this subject^17^ and established induced pluripotent stem cells (iPSC) from the subject.^25^, ^26^, ^27^ Several clinical studies suggest that reduced reelin in the brain and peripheral blood might be associated with mental disorders.^28^, ^29^, ^30^, ^31^

Behavioral changes in *Reln‐*mutant mice, reported by numerous studies, exhibit schizophrenia‐like phenotypes (Table 1)^5^, ^31^, ^32^, ^33^, ^34^; however, insights into the biological mechanisms during brain development due to reelin reduction in humans remain limited. One clue regarding this issue comes from our recent quantitative single‐cell time‐lapse analysis of neuronal differentiation and migration using *Reln*‐del iPSC revealing the developmental significance of reelin in migrating directionality.^25^ To examine directional abnormality in neuronal migration observed in human *RELN*‐deleted iPSC *in vivo*, we generated a mouse line in the C57BL/6J strain harboring the specific *Reln* deletion identified by us in a Japanese subject with schizophrenia, and named the line *Reln*‐del mice. Our behavioral analyses revealed abnormal sociability in heterozygous *Reln*‐del mice. This novel *Reln*‐del mouse model may be valuable for understanding reelin signaling, and for studies on the pathological mechanisms of schizophrenia via the reelin deletion.

**Table 1:** Comparison of behavioral changes in *Reln* mutant mice

	Sawahata *et al*., 2019 (Present study)	Sobue *et al*. (2018)^17^	Lalonde *et al*. (2004^33^	Salinger *et al*. (2003^34^	Qiu *et al*. (2006)^5^	Sakai *et al*. (2016)^31^
Mutant mice strains (Background)	*Reln*‐del (C57BL/6)	Orleans hetero (BALB/C)	Orleans homo (BALB/C)	Jackson hetero (B6C3Fe)	Jackson hetero (B6C3Fe)	⊿C‐KI (C57BL/6)
Age, sex	10–19 weeks, male/female	10–15 weeks, male/female	12 weeks, male	10 weeks, male	6 weeks, male	11 weeks, male
Stationary beams	NA	NA	↓	NA	NA	NA
Acoustic responsiveness	NA	NA	NA	NA	NA	NA
Wire hang latency	NA	NA	NA	NA	NA	↓
Locomotor activity	=	=	↑	NA	NA	NA
Open field	=	↑	↑	=	=	↑
Elevated plus maze	=	=	↓	NA	=	↑
Tail suspension test	NA	NA	NA	NA	NA	=
Porsolt forced swim test	NA	NA	NA	NA	NA	=
Y‐maze	=	=	NA	NA	NA	=
Barnes maze test	NA	NA	NA	NA	NA	=
T‐maze	NA	NA	=	NA	NA	↓ (Working memory)
Novel object recognition	=	=	NA	↓	NA	NA
Social interaction test	↓ (Social novelty)	↓	NA	=	NA	↓
Rotarod test	=	↓	↓	NA	=	↑
Fear conditioning test	=	=	NA	=	↓ (Context)	=
Water maze test	NA	NA	↓	NA	=	NA
Prepulse inhibition	=	↑ (Acoustic response)	NA	=	↓ (82 dB)	=
MK801‐induced hyperlocomotion	NA	=	NA	NA	NA	NA
METH‐induced hyperlocomotion	NA	↓	NA	NA	NA	NA

↑, higher than wild‐type; ↓, lower than wild‐type; =, no difference; NA, not applicable.

## Methods

### CRISPR/Cas plasmid for *Reln* deletion

A pair of oligo DNAs (Invitrogen, Carlsbad, CA, USA) corresponding to *Reln* guide RNA was hybridized and ligated using T4 DNA ligase (TOYOBO, Osaka, Japan) into linearized pSpCas9(BB)‐2A‐GFP (PX458) plasmid (Addgene plasmid # 48138; http://n2t.net/addgene:48138; RRID:Addgene_48138) digested with BbsI (NEB, Ipswich, MA, USA) as previously described.^35^, ^36^, ^37^ DNA primers are listed in Table S2.

### Animal experiments

All research and animal care procedures were approved by the Nagoya University Animal Care and Use Committee. Mice were housed in groups of maximum six animals per cage and maintained on a regular 12‐h light/dark cycle (09:00 to 21:00 hours light period) at a constant 23°C temperature. Food and water were available *ad libitum*. Heterozygous *Reln‐*del mice were generated by intercrossing *Reln‐*del males and C57BL/6J females. Littermate WT C57BL/6J mice were used as controls, and 10–19‐week‐old *Reln‐*del and WT mice were used in all experiments. All animal protocols were approved by the Animal Care and Use Committee of Nagoya University Graduate School of Medicine; in addition, the Principles for the Care and Use of Laboratory Animals, which were approved by the Japanese Pharmacological Society, and the National Institutes of Health Guide for the Care and Use of Laboratory Animals were followed.

Other materials and methods are described in Supplementary materials.

## Results

### Generation of *Reln‐*del mice

**Figure 1:**
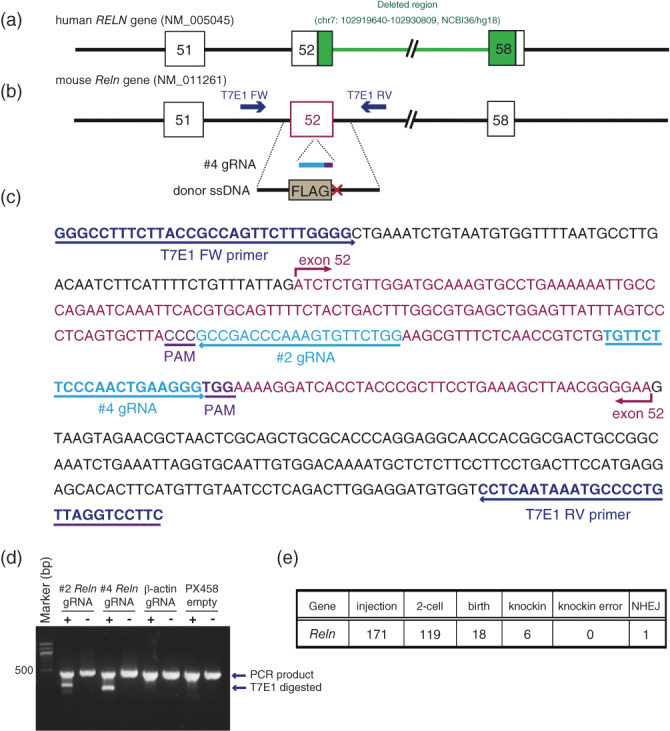
Generation of *Reln*‐del mice by CRISPR/Cas9. Schematic *RELN* gene structure in (a) human and (b) mouse. (a) The genomic region (exon 52 to 58) that was deleted in the Japanese subject with schizophrenia is shown in green.^24^ (b) CRISPR/Cas9 strategy for generating the model mouse with *Reln* deletion as shown in (a). Guide RNAs (gRNA) and single‐stranded DNA (ssDNA) for insertion of a stop codon in exon 52 of *Reln* gene. (c) Sequence around mouse *Reln* exon 52 and design of gRNA for CRISPR target and primers to perform the T7 endonuclease I (T7EI) assay for cleavage activity. (d) Results of the T7EI assay. The amount of shortened DNA shows cleavage activity by T7EI. (e) Summary of the results following injection of the CRISPR mixture into fertilized C57BL/6J egg pronucleus.

We previously identified a schizophrenia subject harboring a partial *RELN* deletion in chr 7 (102919640–102930809, NCBI36/hg18), which includes the region encompassing exons 52–58 encoding reelin repeats 7–8 (Fig. 1a).^17^, ^24^ We generated a genetically modified mouse corresponding to this deletion in *RELN* by genome editing using the CRISPR/Cas9 method; the strain was C57BL/6J, the standard murine line for general behavioral analyses. The *Reln* gene structure between human and mouse is highly conserved. First, we designed 2 CRISPR‐targeted guide RNAs and a donor DNA for inserting a FLAG‐tag sequence and a stop codon in exon 52 to generate the *Reln*‐del mouse model (Fig. 1b,c). When two guide RNAs were transiently transfected into the mouse neuroblastoma cell line Neuro2a, we found that the #4 guide RNA showed the strongest cleavage activity by the T7E1 assay (Fig. 1d). We examined the possibility of the off‐target effect of the #4 guide RNA using the CRISPR Design tool supported by MIT (http://crispr.mit.edu/) and confirmed that there were no pseudo‐sequences due to a mismatch of less than three base pairs in the whole mouse coding regions (Table S1). The #4 guide RNA was chemically synthesized as a Crispr‐RNA, and tracr‐RNA, Cas9 protein, and the donor DNA were co‐microinjected into 171 fertilized eggs in C57BL/6J mice bred at Charles River, which were cultured up to the two‐cell stage. Next, we transplanted 119 eggs into the oviducts of pseudopregnant mice, and 18 littermates were born. As a result, we confirmed that six knock‐in mice were obtained as designed by polymerase chain reaction (PCR) cloning (Fig. 1e).

### Validation of *Reln*‐del mice

**Figure 2:**
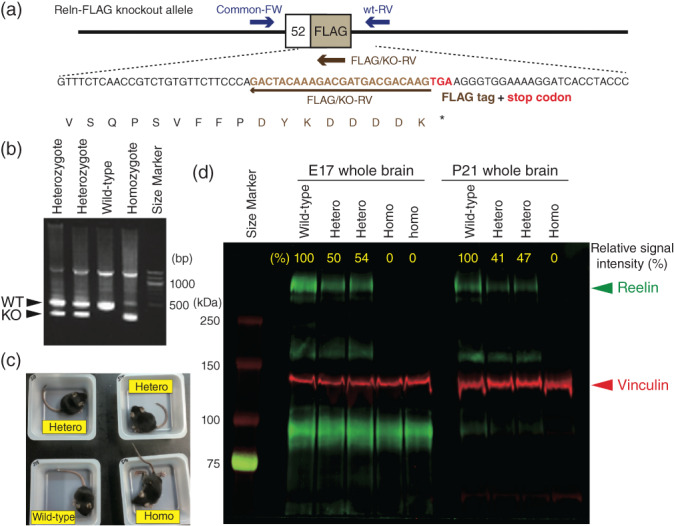
Validation of *Reln*‐del mice. (a) Primer design for genotyping *Reln*‐del mice. (b) Polymerase chain reaction for genotyping *Reln*‐del mice. Larger bands indicate wild‐type (WT) allele, and smaller bands indicate mutant allele. (c) Appearance of *Reln*‐del mouse 21 days after birth. Homozygous *Reln*‐del mice staggered (see also Supplementary Movie S1). (d) Immunoblot analysis to confirm reelin deletion in *Reln*‐del mouse brain. Fifty‐microgram total lysate from the whole brain of postnatal day 21 mice were loaded in each well. The signal was detected by an Odyssey imaging platform. The molecular weight of full‐length reelin protein is approximately 388 kDa, which is indicated by the green signal. The internal control vinculin (117kDa) is indicated by the red signal. The expression level of full‐length reelin relative to that of vinculin was calculated by the software attached to Odyssey and is shown in yellow font.

Screening of the generated mice by conventional genotyping was achieved with three primers designed to distinguish wild‐type (WT) and mutant (*Reln‐*del) alleles by agarose gel electrophoresis (Fig. 2a,b, Table S2). Homozygous *Reln*‐del mice were born normally and exhibited normal growth until 21 days after birth. However, *Reln*‐del mice exhibited phenotypic similarities to *staggerer* and other *reeler* mice (Fig. 2c, Movie S1).

We also validated the reelin protein expression level in the novel *Reln*‐del mice by immunoblotting (Fig. 2d). In embryonic day 17 and postnatal day 21, the expression of reelin in heterozygous *Reln*‐del mice was approximately half the reelin expression in WT mice; reelin was detected barely in homozygous *Reln‐*del mice in those ages. Expression of vinculin as an internal control was comparable between the genotypes. The specific reelin antibody used in the current study could not detect lower molecular‐weight bands by immunoblotting in homozygous *Reln*‐del mice, indicating that they were null mutants with no truncated reelin.

### Histological analysis of *Reln*‐del mice

**Figure 3:**
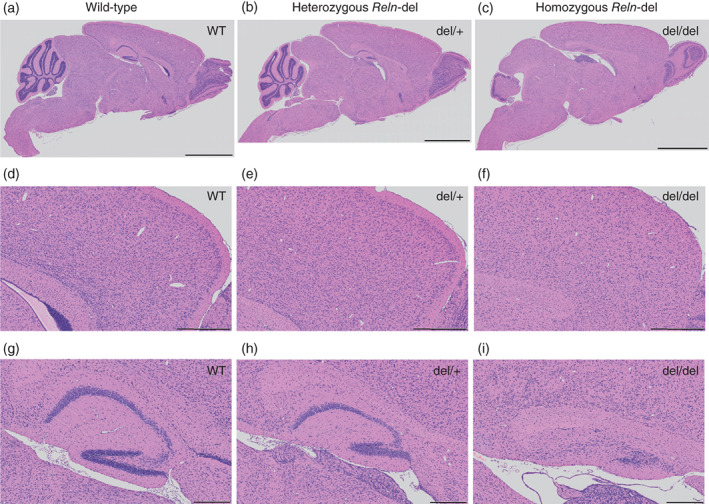
Histological analysis of brain in *Reln*‐del mice. (a–c) Hematoxylin/eosin (HE)‐stained sagittal sections of whole brain in (a) wild‐type (WT) mice, (b) heterozygous *Reln*‐del mice, and (c) homozygous *Reln*‐del mice. Scale bars: 2 mm. (d–f) HE‐stained cortical sections, highly magnified from those presented in (a–c). Scale bars: 500 μm. (g–i) HE‐stained hippocampal sections, highly magnified from those presented in (a–c). Scale bars: 200 μm. All sections are from 2–3‐week‐old mice.

We investigated histological changes in *Reln*‐del mice. Hematoxylin/eosin‐stained brain slices of homozygous *Reln*‐del mice showed severe brain malformation (Fig. 3). Specifically, cerebellar atrophy, enlargement of the cerebral ventricles (Fig. 3a–c) and cerebral dysplasia (Fig. 3d–f), which were one of the typical *reeler* phenotypes^38^, ^39^, ^40^, ^41^ were remarkable. In addition, we observed hippocampal malformation and disruption of the dentate gyrus and granule layer (Fig. 3g–i). On the other hand, heterozygous *Reln*‐del mice did not show severe brain malformation like homozygous *Reln*‐del mice.

### Behavioral analyses of *Reln*‐del mice

*Reln* mutation is a risk factor in several mental disorders, such as schizophrenia and autism spectrum disorder. Therefore, we performed global behavioral analyses using the following 10 behavioral tests to determine whether *Reln*‐del mice were a relevant model for schizophrenia: locomotor, rotarod, open field, elevated plus maze (EPM), social interaction, marble burying, PPI, Y‐maze, novel object recognition, and fear conditioning tests.

We used heterozygous *Reln*‐del mice for all behavioral analyses, because nearly all homozygous *Reln*‐del mice were hypoplastic and had difficulty in reaching the adult stage. Additionally, the original Japanese subject that was the basis of our studies harbored a heterozygous *Reln* deletion.^17^

**Figure 4:**
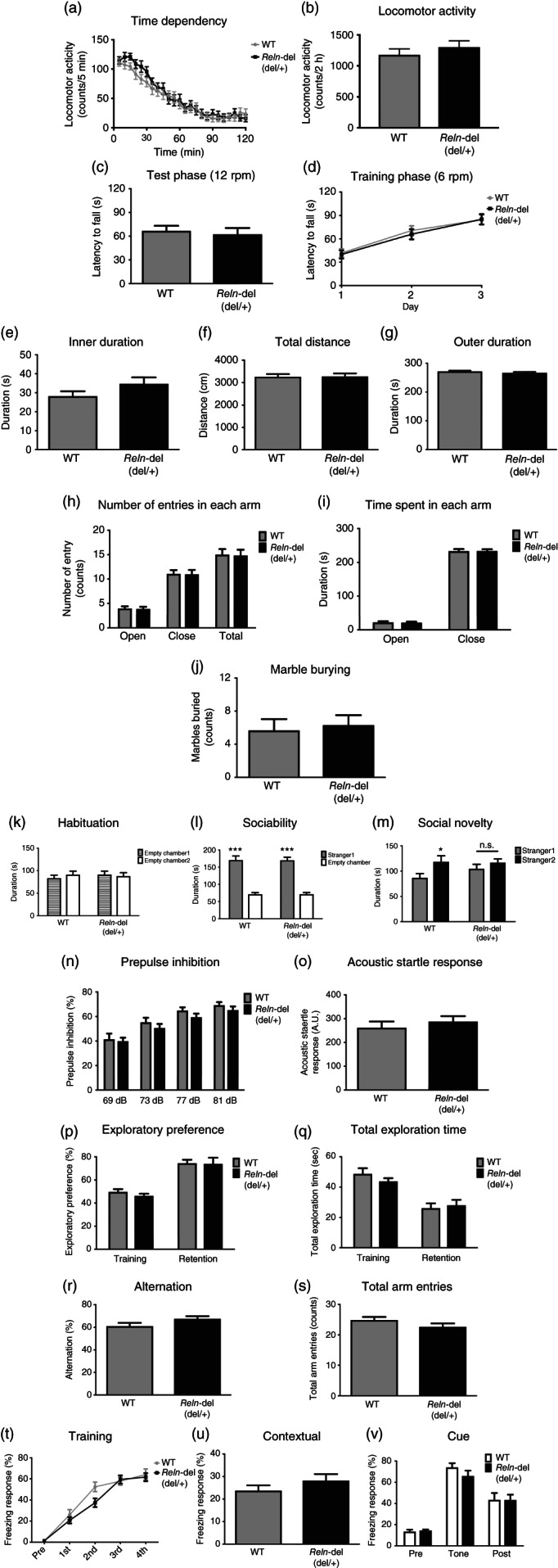
Behavioral analyses in heterozygous *Reln*‐del mice. Performance in the (a,b) locomotor activity, (c,d) rotarod, (e–g) open field, (h,i) elevated plus maze, (j) marble‐burying, (n,o) pre‐pulse inhibition, (p,q) novel object recognition, (r,s) Y‐maze, and (t–v) fear‐conditioning tests. Data represent the mean ± standard error of the mean (SEM; *n* = 24 for wild‐type [WT] mice, *n* = 26 for *Reln‐*del mice in Fig. 4a,b; *n* = 24 for WT mice, *n* = 26 for *Reln*‐del mice in Fig. 4c,d; *n* = 24 for WT mice, *n* = 27 for *Reln‐*del mice in Fig. 4e–g; *n* = 24 for WT mice, *n* = 25 for *Reln*‐del mice in Fig. 4f,i; *n* = 12 for WT mice, *n* = 13 for *Reln‐*del mice in Fig. 4j; *n* = 24 for WT mice, *n* = 26 for *Reln‐*del mice in Fig. 4n,o; *n* = 12 for WT mice, *n* = 13 for *Reln‐*del mice in Fig. 4p,q; *n* = 22 for WT mice, *n* = 22 for *Reln‐*del mice in Fig. 4r,s; *n* = 12 for WT mice, *n* = 13 for *Reln‐*del mice in Fig. 4t–v). (k–m) Performance in the three‐chambered social interaction test: (k) habituation, (l) sociability, and (m) social novelty sessions. Data represent the mean ± SEM (*n* = 18–20 for WT mice, *n* = 20 for *Reln‐*del mice). ***P < 0.001 between empty chamber and stranger 1 or *P < 0.05 between stranger 1 and stranger 2 (two‐way analysis of variance with post‐hoc Sidak's multiple comparisons test).

First, we evaluated the general locomotor function in heterozygous *Reln*‐del mice, because homozygous mice showed severe incoordination and cerebellar atrophy, which may change motor, emotional, and cognitive function. Heterozygous *Reln*‐del mice showed normal locomotor activity and coordinated movement in the locomotor and the rotarod tests (Fig. 4a–d). These data show that we could evaluate emotional and cognitive functions of heterozygous *Reln*‐del mice with normal motor function.

A previous study has reported that heterozygous *reeler* mice have reduced anxiety.^42^ Therefore, we evaluated anxiety in the open field and EPM tests. Typically, WT mice spent more time in the periphery than the center of the circle in the open field test and preferred the closed arms compared with the open arms in the EPM test, given that the center of the circle and the open arm were sources of anxiety in mice. Importantly, there were no significant differences between the WT and *Reln*‐del mice in both tests (Fig. 4e–i). To investigate anxiety and irrational behavior, we performed the marble‐burying test and found that *Reln*‐del mice showed normal behavior (Fig. 4j).

Impairments of social behavior and sensorimotor gating function are the main symptoms of schizophrenia. To examine these points of heterozygous *Reln*‐del mice, we performed the three‐chambered social interaction and the PPI tests. There were no significant differences in habituation or sociability sessions between the WT and heterozygous *Reln*‐del mice (Fig. 4k,l). In the social novelty session, WT mice significantly preferred the novel mouse (stranger 2) than the familiar mouse (stranger 1). Conversely, heterozygous *Reln*‐del mice showed the same interest in both strangers 1 and 2 (Fig. 4m). In the PPI test, heterozygous *Reln*‐del mice exhibited a phenotype similar to that of WT mice (Fig. 4n,o). The difference may have caused low attention to stranger mice, but not anxiety to the strangers and some kind of recognition, as the levels of WT and heterozygous *Reln*‐del mice were not different in open field (Fig. 4e–g), EPM (Fig. 4h,i), or novel object recognition tests (Fig. 4p,q). Further in heterozygous *Reln*‐del mice, *Reln*‐del mice exhibited no significant phenotypes in memory function in the Y‐maze and fear‐conditioning test (Fig. 4r–v).

### Cerebellar granule cell migration in *Reln*‐del mice

**Figure 5:**
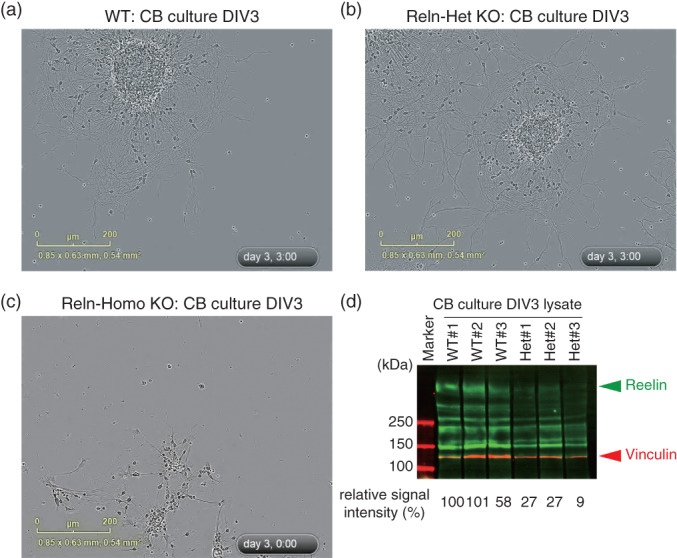
*Reln*‐del cerebellar reaggregation culture. (a–c) Time‐lapse movies of cerebellar reaggregation cultures during days 0–3. Final point of each movie in cultures from (a) wild‐type (WT), (b) heterozygous *Reln*‐del, and (c) homozygous *Reln‐*del mice. Full movies are in [Link], [Link]. (d) Immunoblot analysis of *Reln*‐del cerebellar reaggregation cultures at day 3. The immunoblots were probed with antibodies against reelin.

We performed the time‐lapse analysis to assess migration in cerebellar granule cells isolated from *Reln*‐del mice and conducted biochemical analysis of reelin signaling as well. Reelin binding to its target receptor, such as apolipoprotein E receptor 2 and very‐low‐density‐lipoprotein receptor (VLDLR), leads to the phosphorylation of the adaptor protein Dab1.^43^ We prepared cerebellar granule neurons from postnatal WT and *Reln*‐del mice for reaggregation, which is often used to study neuronal migration,^44^ and conducted a 3‐day time‐lapse analysis (Fig. 5, [Link], [Link]). Then, we performed automatic recognition and quantification of the cell body and neurite length (Fig. S1). When comparing the cerebellar granule neuronal cultures of the WT and heterozygous *Reln*‐del mutant mice, both the average distance covered during neuronal migration from the reaggregates (WT: 97.69 μm [72 cells]; heterozygous *Reln*‐del: 91.87 μm [73 cells]) and the average neurite length (WT: 21.41 μm [10 views]; heterozygous *Reln*‐del: 16.82 μm [10 views]) were not significantly different (Fig. S1); however, reaggregates from homozygous *Reln‐*del mice were affected by cerebellar hypoplasia and showed severe structural defects compared with those from both WT and heterozygous *Reln‐d*el mice; therefore, homozygous *Reln‐*del mice were difficult to quantify (Fig. 5c, Movie S4). Next, we analyzed the reelin signaling pathway in these cerebellar neuronal reaggregation cultures. First, we confirmed that reelin was highly expressed in WT cultures and that the reelin protein expression level was reduced in cultures from heterozygous *Reln*‐del mice (Fig. 5d), whereas the expression levels of the internal control vinculin and the dopaminergic neuronal marker tyrosine hydroxylase were unchanged (Fig. S2a). Finally, we examined the reelin signaling quantitatively by analyzing the relative expression levels of p‐Dab1 and total Dab1 (Figs. S2b,c) and found that the phosphorylation of Dab1 was likely impaired in cultures from heterozygous *Reln*‐del mice.

## Discussion

Disorders of neuronal migration in humans result from the disruption of the normal movement of neurons from their original site of birth to their final destination during early development.^45^ Mutations of many genes are involved in neuronal migration disorders and include *LIS1* and *DCX* in classical lissencephaly spectrum, *TUBA1A* in microlissencephaly with agenesis of the corpus callosum, and *RELN* and VLDLR in lissencephaly with cerebellar hypoplasia.^8^, ^45^ Genetic studies on rare variants implicate that *RELN* is associated with schizophrenia.^19^, ^46^, ^47^ Furthermore, our copy‐number variation analysis identified a subject with schizophrenia who harbored a novel exonic deletion in *RELN*.^24^ As reelin plays important roles in various pathways from development to postnatal function, *Reln‐*del mice are expected to be a reasonable schizophrenia model that can reveal the pathogenic mechanisms underlying schizophrenia. Future studies can facilitate accumulation of sufficient genetic data and clinical phenotypes in humans for elucidation of the molecular mechanisms of schizophrenia caused by a decrease in reelin expression.

In the current study, we generated a novel *Reln*‐del mouse model to mimic the Japanese subject with schizophrenia harboring the specific exonic deletion in *RELN* and compared it with the findings of previous studies on other *reeler*‐associated models, including Orleans *reeler* mice (Table 1). Specifically, we designed a *Reln*‐del mutant allele carrying mutations to code a FLAG‐tag and stop codon in exon 52, corresponding to the amino acid residues within the seventh and eighth reelin repeats (Fig. 1). The homozygous *Reln*‐del mice exhibited the typical phenotypes of the *staggerer* as well as the classical *reeler* mice (Fig. 2c).^38^ Immunoblotting of whole‐brain lysates using an anti‐reelin antibody recognizing the N‐terminal region of reelin revealed that the endogenous reelin level, which was barely detectable in homozygous *Reln*‐del brain lysates, was approximately half of that in WT mice in heterozygous *Reln*‐del mice (Fig. 2d). In contrast, Orleans *reeler* mice have a transcriptional reelin product with a 220‐bp deletion within the seventh and eighth reelin repeats and potentially express a truncated reelin.^11^, ^12^ Our novel *Reln‐*del mice more faithfully reflect the Japanese subject with the *RELN* deletion compared with our previous study using Orleans *reeler* mice.^17^ In addition, we generated the *Reln‐*del mice in the C57BL/6J strain that is commonly used for behavior studies in schizophrenia models, distinct from Orleans *reeler* mice, which were maintained in the Balb/c strain. Differences of inbred strains and/or reelin products might underlie differences in the behavioral phenotypes between the mouse models (Table 1).

Histological analysis showed cerebellar atrophy, hippocampal dysgenesis, and cerebral dysplasia in homozygous *Reln*‐del (Fig. 3). These phenotypes are similar to those of both Jackson and Orleans *reeler* homozygous mice.^38^, ^39^, ^40^, ^41^ On the other hand, we found that heterozygous *Reln*‐del mice did not show severe brain malformation like homozygous *Reln*‐del mice (Fig. 3). These results are similar to those reported by previous other studies.^38^, ^41^

As the results of global behavioral analyses, we found abnormal social novelty in heterozygous *Reln*‐del mice (Fig. 4), indicating that heterozygous *Reln*‐del mice partially mimicked the Japanese subject with schizophrenia who harbors the same *RELN* mutation. Several studies reported that Relnrl‐J/+ mice showed abnormal behaviors, such as PPI deficits,^5^, ^48^ unusual anxiety,^42^ and reduced cognition and sociability.^5^, ^16^ These differences may be associated with the novel *RELN* mutation in heterozygous *Reln*‐del mice. However, the expression pattern of reelin protein in heterozygous *Reln*‐del was similar to that of Relnrl‐J mice based on our immunoblot analysis, which indicate that the reelin‐deficient phenotype might be minimally affected by the growth environment or genetic background. It was reported that reelin related with neuronal plasticity and spine formation.^7^ Most heterozygous reelin mutant mice, including our mice, showed abnormal behaviors without clear histological changes.^5^, ^17^, ^31^, ^33^, ^34^ These kinds of abnormal behaviors may be caused by minimal changes, such as neuronal plasticity and spine formation. Approximately half the levels of reelin may be sufficient for normal neuronal development in the critical developmental stages because reelin signaling includes autoinhibition systems, such as Dab1 reduction. However, reelin is invaluable in the adult stage and has some neuronal function because expression levels are less than those observed in the developmental stage and distribution is different. These phenomena suggest that there is a lack of gene‐dose dependency in mice with *Reln‐*del.

A study in humans showed that patients with lissencephaly carrying *RELN* mutations in splicing recognition sites also exhibited cerebellar hypoplasia due to a decrease in full‐length reelin,^8^ in agreement with the features such as altered neuronal differentiation and migration observed in our *in vitro* phenotypes.^44^ Specifically, we showed severe structural defects in homozygous *Reln*‐del cultures (Fig. 5c and Movie S4), which closely associate with cerebellar hypoplasia,^8^ indicating a potentially reliable lissencephaly model. Conversely, neurons in heterozygous *Reln*‐del cultures are expected to be more likely a good model of schizophrenia based on genetic studies.^17^, ^24^ Neurons in heterozygous *Reln*‐del cultures showed an unmarked phenotype of migration abnormality, but there were no differences in gene dose between neurite migration and elongation *in vitro* (Fig. 5a,b, Fig. S1, and [Link], [Link]).

The iPSC technology is a promising tool for creating a new generation of pathophysiological assays for *in vitro* drug screening, which allows time‐course analysis of neurons from patient‐derived iPSC that are not readily available in human studies, and recent studies utilizing iPSC facilitate the interrogation of molecular pathways and cellular dynamics in humans.^49^ Nonetheless, the pathogenesis of schizophrenia remains poorly understood, and analysis in animal models is necessary for understanding the neural circuits and tissue structures in schizophrenia. Using reliable and predictive mouse models is essential to understand the neurobiological basis of schizophrenia and the development of novel drugs with improved therapeutic efficacy.^50^

Our previous trajectory analysis of the highly homologous neurons, such as tyrosine‐hydroxylase‐positive neurons from iPSC, is one of the best ways to understand the mechanisms of microstructural defects in the brains of subjects with schizophrenia.^25^, ^51^ Cerebellar neurons from homozygous *Reln*‐del mice showed a very severe migration defect that was similar to the outgrowth abnormality of tyrosine‐hydroxylase‐positive neurons derived from iPSC of the Japanese subject with *Reln*‐del. In our *in vitro* analysis, the cell bodies and neurites of the cerebellar reaggregates were too fine, and the population was too dense, which hindered the measurement of the area of each cell body and the trajectory analysis. Overcoming these technical issues will allow for more precise comparisons of neuronal dynamics and morphology in these cultures with those of human iPSC‐derived neurons. *In vitro* analyses alone are insufficient to elucidate the molecular pathology of individuals by behavioral analyses of schizophrenia model mice. Advantages of highly homologous differentiation of human iPSC into specific neuronal subtypes will significantly aid in consideration of *in vitro* analyses, such as reelin signaling during migrating, probably promoting the development of reelin‐related therapies.

In conclusion, our *in vitro* and *in vivo* data indicate that the novel *Reln*‐del mice, using CRISPR/Cas9, which partially mimicked schizophrenia‐like behavior, provide a novel platform for elucidation of the pathogenesis underlying schizophrenia and the development of reelin‐based therapies.

## Disclosure statement

The authors have no conflicts of interest to declare.

## Author contributions

M.S. and D.M. wrote the main text and prepared most of the figures. D.M. generated the *Reln‐*del mice. M.S. performed most of the behavioral and histological experiments. D.M., Y.A., H.K., I.K., K.K., A.S., E.S., M.S., A.K., R.I., B.A., H.K., and K.I. performed molecular and cellular biological experiments. All other authors commented on and refined the manuscript. N.O., K.Y., T.N., T.N., and K.K. supervised the overall project. All authors have carefully read the paper and approved the final manuscript.

## Supporting information


**Table S1.** Mutations in coding regions at putative off‐target loci containing up to 4 bp mismatches for *Reln* target are listed. This list was calculated by following CRISPR design tool: http://crispr.mit.edu/. Mismatches compared to on‐target sequence are shown in red. PAM sequences are labeled in green.
**Table S2.** DNA primers used in this study.

**Figure S1.** Quantitative analysis of cerebellar reaggregation culture (refer to Fig. 5). (a–c) Cell body (green) and neurite (magenta) were automatically recognized by NeuroTrack software from the data captured by time‐lapse imaging with IncuCyte Zoom [(a) WT, (b) heterozygous *Reln‐*del, and (c) homozygous *Reln*‐del]. (d) Measurements of the distance of the cell body (recognized by NeuroTrack) from the aggregate. The migration distances of 72 cells in the WT and 73 cells in the heterozygous *Reln‐*del were measured; no significant difference was found when the measurements were compared by *t*‐test using Prism8 software. In the homozygous *Reln‐*del, the aggregate was hardly formed, and it was therefore impossible to measure any migration distance. (e) The length of neurites recognized by NeuroTrack was quantified. In WT and heterozygous *Reln‐*del, the lengths of neurites in 10 fields were compared by multiple *t*‐tests using Prism8 software and no significant difference was found.

**Figure S2**. *Reln*‐del cerebellar reaggregation culture (refer to Fig. 5). Immunoblot analysis of *Reln*‐del cerebellar reaggregation cultures at Day 3. The immunoblots were probed with antibodies against (a) tyrosine hydroxylase (TH), (b) disabled 1 (DAB1) (pY232), and (c) phospho‐DAB1(p‐DAB) together with antibody against glyceraldehyde 3‐phosphate dehydrogenase as an internal control and quantified by the Odyssey detection system.

**Movie S1.** Behaviors of *Reln*‐del mice (refer to Fig. 2c).

**Movie S2.** Migrations of neurons from WT cerebellar aggregation culture (refer to Fig. 5a).

**Movie S3.** Migrations of neurons from heterozygous *Reln*‐del cerebellar aggregation culture (refer to Fig. 5b).

**Movie S4.** Migrations of neurons from homozygous *Reln*‐del cerebellar aggregation culture (refer to Fig. 5c).

**Appendix S1.** Supporting information.
