# Supplementary material for: Generation and analysis of novel Reln‐deleted mouse model corresponding to exonic Reln deletion in schizophrenia
Source: Psychiatry Clin Neurosci. 2020 Mar 5;74(5):318–27. doi: 10.1111/pcn.12993 (PMC7318658; doi:10.1111/pcn.12993)
Supplement: Supplementary file 8 — Appendix S1. Supporting information. [file PCN-74-318-s008.docx]

**Supporting Information**

**Table of Contents**

**I. Supplementary Methods**

**II. Supplementary Figures and Movies**

**Figure S1.** Quantitative analysis of cerebellar reaggregation culture (refer to Figure 5)

**Figure S2**. Immunoblot analysis *Reln*-del cerebellar reaggregation culture (Refer to Figure 5)

**Supplementary Movie 1.** Behaviors of *Reln*-del mice (Refer to Figure 2c)

**Supplementary Movie 2.** Migrations of neurons from WT cerebellar aggregation culture (Refer to Figure 5a)

**Supplementary Movie 3.** Migrations of neurons from heterozygous *Reln*-del cerebellar aggregation culture (Refer to Figure 5b)

**Supplementary Movie 4.** Migrations of neurons from homozygous *Reln*-del cerebellar aggregation culture (Refer to Figure 5c)

**III. Supplementary Tables**

**Table S1.** Mutations in coding regions at putative off-target loci containing up to 4 bp mismatches for Reln target are listed.

**Table S2.** DNA primers used in this study

**IV. Supplementary References**

**I. Supplementary Methods**

**T7 endonuclease I assay**

T7 endonuclease I (T7EI) assay using mouse Neuro2a cells was performed as described previously. ^1^ Briefly, *Reln-*pX458 or empty pX458 plasmid was transfected into Neuro2a cells in a 12-well plate using Lipofectamine 3000 (Life Technologies). At 48 hours post-transfection, genomic DNA was prepared by proteinase K (P2308, Sigma-Aldrich, USA) digestion and ethanol precipitation. Next, *Reln* loci were amplified with PCR from the purified genomic DNA with specific primers (Supplementary Table 2). The PCR products were slowly annealed and digested with the T7EI enzyme at 37°C for 30 minutes and analysed by electrophoresis in 2% agarose gel.

**Chemical synthesis of crRNA and tracrRNA**

*Reln*-CRISPR-RNA (crRNA) (5’-UGUUCUUCCCAACUGAAGGGguuuuagagcuaugcuguuuug

-3’) and transactivating RNA (tracrRNA) (5’- AAACAGCAUAGCAAGUUAAAAUAAGGCUAGUCCGUUAUCAACUUGAAAAAGUGGCACCGAGUCGGUGCU-3’) were chemically synthesized and purified by polyacrylamide gel electrophoresis (Fasmac, Atsugi, Kanagawa, Japan).

**Injection**

Mixtures for injection were prepared according to a previously defined method. ^2^ To develop *Reln*-del mouse, Cas9 proteins, *Reln*-crRNA and tracrRNA and donor single-strand DNA (ssDNA) were mixed in 0.1 M Tris-EDTA buffer to working concentrations of 100 ng/μl, 0.61 pmol/μl, 0.61 pmol/μl, and 10 ng/μl, respectively. Cas9 proteins were purchased from NEB (catalog # M0386S, USA). Donor ssDNA, coding FLAG-tag and stop codon and targeting exon 52 of mouse *Reln*, were chemically synthesized by Fasmac (Japan). The mixture was incubated at 37°C for at least 15 minutes and injected into pronuclei of one-cell-stage zygotes obtained from C57BL/6J mice (Charles River, USA).

**PCR screening and genotyping**

For PCR screening of *Reln-*del embryos born from the zygotes injected with CRISPR mix, genomic DNA was prepared from tails by proteinase K digestion and subsequent phenol extraction using standard methods. *Reln-*del mice were screened by PCR with KOD FX Neo (TOYOBO) and analysed by electrophoresis in 2% agarose gel. PCR primers are listed in Supplementary Table 1. PCR products were further cloned with the Zero Blunt TOPO PCR cloning kit (Life Technologies, USA) and analysed by sequencing as described previously.^2^ Conventional screening after establishment of the mouse line was performed by PCR using the mixture of 3 primers listed in Supplementary Table 2.

**Immunoblot analysis**

For immunoblotting to detect reelin, whole-brain extracts were lysed with sodium dodecyl sulphate (SDS) lysis buffer (4% SDS, 20% glycerol and 50 mM Tris-HCl pH 6.8) and sonicated on ice. The lysates were diluted to 5 μg/μl. For loading, equal volumes of 0.01% bromophenol blue and 1M 2-mercaptoethanol were added to the extracts, which were not heat-denatured. A total of 50 μg non-denatured protein was loaded to each well of 5%–20% SDS gradient polyacrylamide gels (e-PAGEL, ATTO, Japan) and transferred to nitrocellulose membranes (Protran, Whatman). The membranes were probed with antibodies against reelin (mouse monoclonal, MAB5366, clone 142, Merck) and vinculin (rabbit polyclonal, #13901, Cell Signalling), followed by incubation with appropriate donkey secondary antibodies (IRDye 680RD and 800CW; LI-COR Biosciences). An infra-red imager was used for the detection and quantification of signal intensities (Odyssey, LI-COR Biosciences). Digital data were exported in TIFF format.

To calculate the ratio of phosphorylated disabled 1 (DAB1, p-DAB1) to total DAB1 by immunoblotting, lysates of 3-day-old cerebellar cultures prepared as stated above were analysed by immunoblotting using anti-p-DAB1 and anti-DAB1 antibodies (rabbit polyclonal, Cell Signalling) as well as an antibody against glyceraldehyde 3-phosphate dehydrogenase (mouse monoclonal, MBL) as an internal control.

**Histological analysis**

Paraffin-embedded brain tissue sections (5 μm) were deparaffinized and placed in hematoxylin in distilled water for 10 minutes. The sections were rinsed in running tap water, dipped in hot water at 50ºC to remove overstaining, stained with eosin for 2 minutes, dehydrated and mounted.

**Behavioural analyses**

Behavioural tests were performed in the following order: open field, Y-maze, EPM, locomotor activity, novel object recognition, social interaction, rotarod test, PPI, fear conditioning and marble burying tests.

Three-chambered social interaction test: The social interaction test was conducted as described previously,^3^ with minor modifications. The apparatus consists of a black Plexiglas rectangular box (52 cm long × 25 cm wide × 23 cm tall) divided into 3 interconnected chambers. The 2 end chambers were equal size (19 cm × 25 cm), and the middle chamber was smaller (12 cm × 25 cm). All sessions were conducted under conditions of illumination (15 lx). In the habituation phase, a test mouse was placed in the entire social test box and allowed to explore for 10 minutes. In the sociability session, an unfamiliar C57BL/6J mouse (stranger 1) that had no prior contact with the subject mouse was placed by the identical clear Plexiglas cylinder (8 cm in diameter, 12 cm tall, with multiple small holes) in one of the side chambers. The test mouse was allowed to explore the entire social test box for a 10-minute session. In the social novelty session, a second, unfamiliar mouse (stranger 2) was placed in the chamber on the other side of stranger 1. Measurements included the amount of time spent around the cylinder by the EthoVision automated tracking program (Noldus, Wageningen, Netherlands).

PPI test. The PPI test was performed as described previously. ^4, 5^ Briefly, after being placed in the chamber under moderately bright light conditions (180 lx) (San Diego Instruments, San Diego, CA), the animals were allowed to habituate for 10 minutes, during which a 65-dB background white noise was present. The animals then received 10 startle trials, 10 no-stimulus trials and 40 PPI trials. The inter-trial interval was between 10 and 20 seconds, and the total session lasted 17 minutes. The startle trial comprised a single 120-dB white noise burst lasting 40 ms. PPI trials comprised a pre-pulse (20-ms burst of white noise at an intensity of 69, 73, 77, or 81 dB) followed, 100 ms later, by the startle stimulus (120 dB, 40 ms white noise). Each of the 4 PPI trials (69, 73, 77, or 81 dB) was presented 10 times. Sixty different trials were presented pseudo-randomly, ensuring that each trial was presented 10 times and that no 2 consecutive trials were identical. The resulting movement of the animal in the startle chamber was measured for 100 ms after the onset of the startle stimulus (sampling frequency, 1 kHz), rectiﬁed, ampliﬁed and fed into a computer, which calculated the maximal response over the 100-ms period. Basal startle amplitude was defined as the mean amplitude of 10 startle trials. PPI was calculated according to the formula: 100 × [1 − (PPx/P120)]% in which PPx was the mean of 10 PPI trials (PP69, PP73, PP75, or PP80), and P120 was the basal startle amplitude.

Locomotor activity: For the measurement of locomotor activity, mice were placed individually in a transparent acrylic cage with a black frosted Plexiglas floor (25 × 25 × 20 cm), and locomotor activity was measured every 5 minutes for 120 minutes using digital counters with an infra-red sensor (BrainScience Idea, Osaka, Japan) under conditions of illumination (15 lx).

Rotarod test: The rotarod test was performed according to a previous study, ^3, 6^ with minor modifications. Both training and test phases were performed under conditions of illumination (15 lx). Mice were trained for 3 consecutive days. During the training phase, mice were placed on a rod rotating at 6 revolutions per minutes, and the time it took to fall from the rod was measured. If a mouse stayed on the rod until the end of the 2-minute trial, the time was recorded as 120 seconds. The test phase was performed after consecutive training. Mice were placed on a rod rotating at 12 revolutions per minute, and the time taken for them to fall from the rod was measured. Each mouse was subjected to 6 trials per day with a 15-minute inter-trial interval in the training and test phases. We calculated the average value for each set of measurements. The apparatus was routinely cleaned with ethanol following each session.

Open field test: Mice were placed at the centre of an open field (diameter, 60 cm; height, 35 cm) under moderate light conditions (85 lx) and allowed to explore it for 5 minutes. The movement of mice was measured automatically using the EthoVision automated tracking program via a camera mounted above the open field.^7^ Measurements included duration in the inner and outer sections and total distance. The open field was divided into an inner circle (diameter, 40 cm) and an outer area surrounding the inner circle.

EPM test. The EPM was constructed and conducted as previously described^7, 8^ with minor modifications. The apparatus was made of plastic material and elevated to a height of 50 cm above the ground. Each arm of the EPM was 16 cm in length and 10 cm in width. Additionally, the closed arms had wall enclosures that were 20 cm high. The central platform was a square of 10 × 10 cm. Mice were allowed to explore in the EPM for 5 minutes under moderate light conditions (100–120 lx). The number of entries and time spent in the open and closed arms were recorded over the entire duration of the test.

Y-maze test: The Y-maze test was performed as described previously.^7, 9^ Each arm was 40 cm long and 12 cm high, 3 cm wide at the bottom, and 10 cm wide at the top. The arms converged in an equilateral triangular central area that was 4 cm at its longest axis. Each mouse was placed individually at the centre of the apparatus and allowed to move freely through the maze during an 8-minute session under conditions of illumination (35 lx). The series of arm entries were recorded visually. Alternation defined as successive entries into the three arms in overlapping triplet sets (e.g. ABC, CAB, BCA, but not BAC) was associated with the capacity of short-term memory. Percent alternation was calculated as the ratio of actual to possible alternations (defined as the total number of arm entries minus 2) multiplied by 100 as shown by the following equation: % alternation = (number of alternations)/(total arm entries–2) × 100.

Novel object recognition test: The novel object recognition test was performed as described previously^7^ with minor modifications. Mice were individually habituated to an open-box (30 × 30 × 35 cm) for 3 days. During the training session, 2 novel objects were placed in the open ﬁeld, and animals were allowed to explore for 10 minutes under moderate light conditions (15 lx). In test sessions, animals were placed back into the same box 24 hours after the training session, one of the familiar objects used during training was replaced by a novel object, and mice were allowed to explore freely for 5 minutes. The preference index was calculated as the ratio of the amount of time spent exploring one of the objects (novel object in test session) over the total time spent exploring both objects and was used to measure cognitive function.

Fear conditioning test: To examine contextual memory, context-dependent conditioned fear test was used according to a previous report.^5^ In the conditioning phase, each mouse was placed in the training cage (30 cm × 30 cm × 40 cm) equipped with a metal floor, and a 15-second tone (85 dB) was delivered (conditioned stimulus). During the last 5 seconds of the tone stimulus, a foot shock of 0.8 mA through a shock generator was delivered as an unconditioned stimulus. This procedure was repeated 4 times with 15-second intervals. Context-dependent test was performed 24 hours after the conditioning. For the context-dependent test, the mouse was placed in the training cage and the freezing response was measured for 2 minutes in the absence of the conditioned stimulus. Tone-dependent test was performed 4 hours after the context-dependent test. Specifically, the freezing response was measured in the neutral cage for 1 minute in the presence of a continuous-tone stimulus identical to the conditioned stimulus using mice that were evaluated in the context-dependent test.

Marble burying test: The marble burying test was performed as described previously.^10,11^ One day prior to testing, mice were individually housed in clean plastic cages (26 cm × 21 cm × 15 cm) with bedding material (sawdust at a depth of 3 cm). On the test day, 12 glass marbles were placed with even spacing on the flattened sawdust on one side of the cage. Mice were placed into a corner of the cage containing the marbles and allowed to explore the cage undisturbed for 15 minutes (40 lx). Marble burying behaviour was counted as a marble with two-thirds of its surface area covered by bedding.

**Statistical analysis**

All data were shown as means ± standard error of the mean and analysed by Prism 7 (GraphPad software, USA). Data from the behavioural tests including three-chambered social interaction, PPI, locomotor, rotarod, EPM, novel object recognition and fear conditioning tests were analysed by two-way analysis of variance with or without repeated measures followed by *post hoc* Bonferroni test. Data from the PPI (acoustic startle response), locomotor (locomotor activity), rotarod (test phase), open field, marble burying, Y-maze and fear conditioning (contextual) tests were assessed using the Mann–Whitney *U* test.

**Cerebellar reaggregate cultures and time-lapse analysis**

Reaggregate cultures were prepared as defined in previous studies.^12-15^ Briefly, cerebellar granule neurones were dissociated from 6-day-old mice. Reaggregates were transferred to plates coated with poly-L-ornithine (Sigma-Aldrich). Time-lapse movies were captured using the IncuCyte live-imaging system (Essen Bioscience, USA).^16^ The images were analyzed with NeuroTrack equipped with IncuCyte Zoom; the cell bodies and neurites migrated from reaggregates were automatically recognized. Regarding the migrated distance, the shortest distance to the aggregate was measured manually on the images for each cell^12^ and the results were directly quantified by NeuroTrack for neurites. Since homozygous *Reln-*del hardly formed reaggregates, statistical analysis using multiple t-test with Prism 8 software was performed between the two groups: WT and hetero *Reln-*del.

**II. Supplementary Figures and Movies**

**Supplementary Figure 1 Quantitative analysis of cerebellar reaggregation culture (refer to Figure 5)**

(a-c) Cell body (green) and neurite (magenta) were automatically recognized by NeuroTrack software from the data captured by time-lapse imaging with Incucyte Zoom [(a) WT, (b) heterozygous *Reln-*del, and (c) homozygous *Reln*-Del]. (d) Measurements of the distance of the cell body (recognized by Neurotrack) from the aggregate. The migration distances of 72 cells in the WT and 73 cells in the heterozygous *Reln-*del were measured; no significant difference was found when the measurements were compared by t-test using Prism8 software. In the homozygous *Reln-*del, the aggregate was hardly formed, and it was therefore impossible to measure any migration distance. (e) The length of neurites recognized by Neurotrack was quantified. In WT and heterozygous *Reln-*del, the lengths of neurites in 10 fields were compared by multiple t-tests using Prism8 software and no significant difference was found.

**Supplementary Figure 2 *Reln*-del cerebellar reaggregation culture (Refer to Figure 5)**

Immunoblot analysis of *Reln*-del cerebellar reaggregation cultures at day 3. The immunoblots were probed with antibodies against tyrosine hydroxylase (TH) (a), disabled 1 (DAB1) (pY232) (b) and phospho-DAB1(p-DAB) (c) together with antibody against glyceraldehyde 3-phosphate dehydrogenase as an internal control and quantified by the Odyssey detection system.

**Supplementary Movie 1.** Behaviors of *Reln*-del mice (Refer to Figure 2c)

**Supplementary Movie 2.** Migrations of neurons from WT cerebellar aggregation culture (Refer to Figure 5a)

**Supplementary Movie 3.** Migrations of neurons from heterozygous *Reln*-del cerebellar aggregation culture (Refer to Figure 5b)

**Supplementary Movie 4.** Migrations of neurons from homozygous *Reln*-del cerebellar aggregation culture (Refer to Figure 5c)

**III. Supplementary Tables**

| **Supplementary Table 1** Mutations in coding regions at putative off-target loci containing up to 4 bp mismatches for *Reln* target are listed. This list was calculated by following CRISPR design tool: http://crispr.mit.edu/. Mismatches compared to on-target sequence are shown in red. PAM sequences are labeled in green. | | | |  |
| --- | --- | --- | --- | --- |
|  |  |  |  |  |
| **sequence 5' to 3'** | **score** | **mismatches** | **UCSC gene** | **locus** |
| TGTTCTTCCCAACTGAAGGG**TGG** | **63** | mReln #4 (on target) | NM_011261.2 | chr5:+21424983 |
|  |  |  |  |  |
| TGTTCTTCCCAAC**A**GAA**T**G**TTGG** | 0 | 3MMs [14:18:20] | NM_175370 | chr1:+58752876 |
| TGT**G**CTTCCC**CT**CTGAA**T**GG**AAG** | 0.1 | 4MMs [4:11:12:18] | NM_001079686 | chr10:-5191977 |
| T**A**TTCTT**GA**CA**T**CTGAAGGG**GGG** | 0.4 | 4MMs [2:8:9:12] | NM_146020 | chr11:-70333080 |
| **A**GTTC**C**TCCC**G**ACTGAAGG**CGAG** | 0.2 | 4MMs [1:6:11:20] | NM_021493 | chr11:-97363221 |
| TGT**G**CTTCCCA**C**CTG**G**AG**A**G**TGG** | 0 | 4MMs [4:12:16:19] | NM_145354 | chr13:+69754455 |
| TG**AC**CTTCCCAA**AA**GAAGGG**GGG** | 0.1 | 4MMs [3:4:13:14] | NM_183208 | chr14:+26481055 |
| T**CC**TCTTCC**G**A**G**CTGAAGGG**AGG** | 0.6 | 4MMs [2:3:10:12] | NM_025943 | chr14:+119282761 |
| **CC**TTC**A**TCCCAA**G**TGAAGGG**AAG** | 0.4 | 4MMs [1:2:6:13] | NM_001163189 | chr15:+80344507 |
| TGT**C**CTTCCCAAC**AAG**AGGG**CAG** | 0 | 4MMs [4:14:15:16] | NM_177821 | chr15:-81479111 |
| TGTTCTT**TTA**AACTGAAGG**AAAG** | 0.4 | 4MMs [8:9:10:20] | NM_019817 | chr15:-103128242 |
| TGTTCT**C**CCCA**CT**TGAAG**T**G**AAG** | 0.1 | 4MMs [7:12:13:19] | NM_172132 | chr17:-56535998 |
| TGTTC**CA**CCCAACT**C**AAG**A**G**TGG** | 0.1 | 4MMs [6:7:15:19] | NM_030749 | chr18:-35577722 |
| **A**GTTCTTCC**A**AACTGAAC**TGCAG** | 0.1 | 4MMs [1:10:18:19] | NM_053135 | chr18:+37571868 |
| **A**GTTCTTCC**A**AACTGAAC**TGCAG** | 0.1 | 4MMs [1:10:18:19] | NM_053136 | chr18:+37581614 |
| **A**GTTCTTCC**A**AACTGAAC**TGCAG** | 0.1 | 4MMs [1:10:18:19] | NM_053137 | chr18:+37595777 |
| T**C**TTCT**G**CCCA**G**C**A**GAAGGG**AAG** | 0.1 | 4MMs [2:7:12:14] | NM_001164420 | chr18:-80489144 |
| TGT**A**CTT**GT**CAACTG**C**AGGG**GGG** | 0.2 | 4MMs [4:8:9:16] | NM_054048 | chr19:-7345028 |
| TGTTCTTC**ATCT**CTGAAGGG**AGG** | 0.2 | 4MMs [9:10:11:12] | NM_001282085 | chr2:-126432929 |
| **G**GTTCTT**T**CCA**T**CTGAA**A**GG**AGG** | 0.2 | 4MMs [1:8:12:18] | NM_177782 | chr2:+166402192 |
| TGTT**G**T**G**CCCAACTGA**T**GG**ACAG** | 0.2 | 4MMs [5:7:17:20] | NM_026651 | chr4:+115832287 |
| T**A**TTCTTC**A**CA**T**CTGAA**T**GG**GGG** | 0.1 | 4MMs [2:9:12:18] | NM_001005788 | chr4:+120604098 |
| **GT**TTCTTCCCAA**A**TGA**T**GGG**AGG** | 0.2 | 4MMs [1:2:13:17] | NM_011284 | chr4:+132332315 |
| TGTTCTTCCCAACT**T**AAG**AACAG** | 0.1 | 3MMs [15:19:20] | NM_001038999 | chr5:+68011547 |
| TGTT**TC**TCCCA**G**CTG**G**AGGG**TGG** | 0.1 | 4MMs [5:6:12:16] | NM_007925 | chr5:-135194971 |
| TGT**G**CTTCCC**C**ACTGA**G**G**A**G**GAG** | 0.1 | 4MMs [4:11:17:19] | NM_175521 | chr5:+138179482 |
| TGTTC**A**TCCCA**TG**T**A**AAGGG**AGG** | 0 | 4MMs [6:12:13:15] | NM_001034863 | chr9:-42918974 |
| TG**AG**CTTCCCAAC**A**GA**T**GGG**AAG** | 0.1 | 4MMs [3:4:14:17] | NM_173413 | chr9:+66693975 |
| T**C**TTCTT**T**CCA**TT**TGAAGGG**CGG** | 0.3 | 4MMs [2:8:12:13] | NM_007492 | chrX:-90537298 |

| **Supplementary Table 2** DNA primers used in this study. | | |  |
| --- | --- | --- | --- |
|  |  |  | |
| PX458-gRNA construction | 5' to 3' sequence |  |  |
| mReln #2 gRNA | Forward | caccGCCAGAACACTTTGGGTCGGC | |
|  | Reverse | aaacGCCGACCCAAAGTGTTCTGGC | |
| mReln #4 gRNA | Forward | caccGCCAGAACACTTTGGGTCGGC | |
|  | Reverse | aaacGCCGACCCAAAGTGTTCTGGC | |
| β-actin gRNA (positive control) | Forward | caccGCATTATGAGTCCTTAAGTGA | |
|  | Reverse | aaacTCACTTAAGGACTCATAATGC | |
|  |  |  | |
| T7E1 assay |  | 5' to 3' sequence | |
| mReln | Forward | GGGCCTTTCTTACCGCCAGTTCTTTGGGG | |
|  | Reverse | GAAGGACCTAACAGGGGCATTTATTGAGG | |
| β-actin | Forward | GACTGGGATCATTTGTTCACACATGCAG | |
|  | Reverse | GGCCCTTGAACTTCGGTATCTACACTGAG | |
|  |  |  | |
| Genotyping |  | 5' to 3' sequence | |
| mReln | Common Forward | GGTGGTAGACATCAAGGGACCTGGG | |
|  | FLAG/KO Reverse | CTTGTCGTCATCGTCTTTGTAGTC | |
|  | Wildtype Reverse | GTGTGCTCCTCATGGAAGTCAGGAAGG | |

**IV. References in supplementary information**

1. Vouillot L, Thelie A, Pollet N. Comparison of T7E1 and surveyor mismatch cleavage assays to detect mutations triggered by engineered nucleases. *G3 (Bethesda)* 2015; **5**: 407-15.

2. Aida T, Chiyo K, Usami T et al. Cloning-free CRISPR/Cas system facilitates functional cassette knock-in in mice. *Genome Biol* 2015; **16**: 87.

3. Sobue A, Kushima I, Nagai T et al. Genetic and animal model analyses reveal the pathogenic role of a novel deletion of RELN in schizophrenia. *Sci Rep* 2018; **8**: 13046.

4. Ibi D, Nagai T, Kitahara Y et al. Neonatal polyI:C treatment in mice results in schizophrenia-like behavioral and neurochemical abnormalities in adulthood. *Neurosci Res* 2009; **64**: 297-305.

5. Nakai T, Nagai T, Tanaka M et al. Girdin phosphorylation is crucial for synaptic plasticity and memory: a potential role in the interaction of BDNF/TrkB/Akt signaling with NMDA receptor. *J Neurosci* 2014; **34**: 14995-5008.

6. Okun E, Barak B, Saada-Madar R et al. Evidence for a developmental role for TLR4 in learning and memory. *PLoS One* 2012; **7**: e47522.

7. Sobue A, Ito N, Nagai T et al. Astroglial major histocompatibility complex class I following immune activation leads to behavioral and neuropathological changes. *Glia* 2018; **66**: 1034-1052.

8. Aoyama Y, Toriumi K, Mouri A et al. Prenatal Nicotine Exposure Impairs the Proliferation of Neuronal Progenitors, Leading to Fewer Glutamatergic Neurons in the Medial Prefrontal Cortex. *Neuropsychopharmacology* 2016; **41**: 578-89.

9. Ibi D, Nagai T, Koike H et al. Combined effect of neonatal immune activation and mutant DISC1 on phenotypic changes in adulthood. *Behav Brain Res* 2010; **206**: 32-7.

10. Alkam T, Kim HC, Hiramatsu M et al. Evaluation of emotional behaviors in young offspring of C57BL/6J mice after gestational and/or perinatal exposure to nicotine in six different time-windows. *Behav Brain Res* 2013; **239**: 80-9.

11. Wulaer B, Nagai T, Sobue A et al. Repetitive and compulsive-like behaviors lead to cognitive dysfunction in Disc1(Delta2-3/Delta2-3) mice. *Genes Brain Behav* 2018; **17**: e12478.

12. Toyo-Oka K, Sasaki S, Yano Y et al. Recruitment of katanin p60 by phosphorylated NDEL1, an LIS1 interacting protein, is essential for mitotic cell division and neuronal migration. *Hum Mol Genet* 2005; **14**: 3113-28.

13. Yamasaki T, Kawaji K, Ono K et al. Pax6 regulates granule cell polarization during parallel fiber formation in the developing cerebellum. *Development* 2001; **128**: 3133-44.

14. Kawaji K, Umeshima H, Eiraku M, Hirano T, Kengaku M. Dual phases of migration of cerebellar granule cells guided by axonal and dendritic leading processes. *Mol Cell Neurosci* 2004; **25**: 228-40.

15. Asou H, Miura M, Kobayashi M, Uyemura K, Itoh K. Cell adhesion molecule L1 guides cell migration in primary reaggregation cultures of mouse cerebellar cells. *Neurosci Lett* 1992; **144**: 221-4.

16. Arioka Y, Shishido E, Kubo H et al. Single-cell trajectory analysis of human homogenous neurons carrying a rare RELN variant. *Transl Psychiatry* 2018; **8**: 129.
